# Fish Need You

**DOI:** 10.1371/journal.pbio.0030075

**Published:** 2005-01-11

There is little in biology that compares in beauty and limpidity to the development of a zebrafish embryo as viewed through a light microscope. The transparent eggshell and embryo tissues expose the minutest details of cell migrations and organ assembly to the curious viewer. Within a day, distinct vertebrate features emerge: a distinct head with the outlines of two large eyes, a quickly pumping heart, a notochord, and a growing array of somites—the bone and muscle precursors—stretching from trunk into tapering tail.

The transparent zebrafish embryo has allowed geneticists to discover a large number of mutants with anomalies in the development of external and internal organs. Seven mutations, collectively known as “You-class,” turn the pointed, chevron-like somites into shallow, rounded arcs (“You” stands for “U-shaped”). Ian Woods and William Talbot now show that the You mutation disrupts a new modulator of Hedgehog signaling.

Hedgehog is an extracellular signaling protein that can impose various fates on target cells at close proximity or over longer distances. Much research is focused on understanding the factors that promote or limit Hedgehog's activity and range. Woods and Talbot propose that the You protein acts in the extracellular environment to promote Hedgehog signaling.

Hedgehog was originally named for mutations that cause excess brush-like denticles to grow on the surface of fruitfly embryos, but it is now known to direct countless developmental decisions in invertebrates and vertebrates alike. In addition, several cancers are known to result from inappropriate Hedgehog signaling. In fish, Hedgehog's best-documented role is in muscle development. In the absence of Hedgehog signaling, cells destined to become slow muscle fibers fail to differentiate properly. A subset of these slow muscle cells—the muscle pioneers—congregate near the dorso-ventral midline of the embryo, where the dorsal and ventral halves of somites converge. When these specialized cells are absent, abnormal somite assembly leads to the U-shaped phenotype.

The authors found that You mutants showed many telltale signs of reduced Hedgehog signaling. Proteins that are normally expressed at certain times during the development of slow muscle cells were not activated in You mutants, indicating that these cells did not form. Mutant embryos also displayed reduced expression of the Hedgehog receptor Patched, a universal reporter of Hedgehog signaling activity. In addition, You mutants had specific ventral spinal chord defects that are shared by known Hedgehog pathway mutants. Yet You mutants expressed Hedgehog normally. Moreover, Hedgehog targets could still be activated in You mutants in response to excess Hedgehog signaling, suggesting that the signaling cascade is left intact. The authors concluded that the You protein was a facilitator rather than a crucial transmitter in Hedgehog signaling, likely acting at a step upstream of a cell's response to Hedgehog. Normal muscle pioneers could form in chimeric embryos (embryos made of wild-type and You mutant cells) regardless of which cells—the Hedgehog-producing cells or Hedgehog-responding muscle precursors—expressed You. This made it most likely that the You protein acted outside the cells, perhaps as a cell matrix component.

The authors mapped the You mutation and found that it disrupted the coding region of a gene encoding a putative secreted protein. The predicted You protein is closely related to members of the mouse SCUBE family, a group of proteins that are defined by characteristic extracellular motifs (although these proteins have not yet been linked to Hedgehog signaling). This observation strengthens the hypothesis that the You protein has extracellular functions, and the researchers' experimental evidence supports a role for You in transport or stabilization of Hedgehog. At later stages You could also participate in other signaling pathways, as its expression does not always coincide with that of Hedgehog during zebrafish development.

